# A urine-based Exosomal gene expression test stratifies risk of high-grade prostate Cancer in men with prior negative prostate biopsy undergoing repeat biopsy

**DOI:** 10.1186/s12894-020-00712-4

**Published:** 2020-09-01

**Authors:** James McKiernan, Mikkel Noerholm, Vasisht Tadigotla, Sonia Kumar, Phillipp Torkler, Grannum Sant, Jason Alter, Michael J. Donovan, Johan Skog

**Affiliations:** 1 Columbia University Medical Center, New York City, NY USA; 2 Exosome Diagnostics, a Bio-Techne Brand, Waltham, MA USA; 3 Icahn School of Medicine at Mt. Sinai, New York City, NY USA

**Keywords:** Prostate Cancer, Exosomes, Urine, Early detection, Prostate biopsy

## Abstract

**Background:**

Initial prostate biopsy often fails to identify prostate cancer resulting in patient anxiety, especially when clinical features such as prostate specific antigen (PSA) remain elevated, leading to the need for repeat biopsies. Prostate biomarker tests, such as the ExoDx™ Prostate *(IntelliScore)*, or EPI test, have been shown to provide individualized risk assessment of clinically significant prostate cancer at initial biopsy; however, the performance in the repeat biopsy setting is not well established.

**Methods:**

As part of a previous prospective clinical validation study evaluating the performance of the EPI test, we collected first-catch, non-DRE urine samples across 22 sites from men with at least one prior negative biopsy scheduled to undergo a repeat prostate biopsy to rule out prostate cancer. All men were 50 years or older with a PSA 2–10 ng/mL. Exosomal mRNA was extracted and expression of three genomic markers, PCA3, ERG and SPDEF was measured. The resulting EPI score was correlated with biopsy results.

**Results:**

229 men with a prior negative biopsy underwent repeat biopsies. ExoDx Prostate demonstrated good performance ruling out high-grade (Grade group 2, GG2, or higher) prostate cancer (HGPCa) using the previously validated 15.6 cut point in the initial biopsy setting. The EPI test yielded an NPV of 92% independent of other clinical features and would have avoided 26% of unnecessary biopsies while missing only five patients with HGPCa (2.1%). Furthermore, the EPI test provided additional information at a cut-point of 20 and 29.6 with an NPV of 94%, potentially delaying 35 and 61% of unnecessary biopsies, respectively. AUC curves and Net Health Benefit Analyses demonstrated superior performance of ExoDx Prostate over PSA and clinical only risk calculators, i.e. ERSPC.

**Conclusions:**

The EPI test provided good performance using the 15.6 cut-point for ruling out HGPCa / GG2 or higher in men undergoing a repeat prostate biopsy with a PSA of 2–10 ng/ml. Furthermore, the test utilizes gene expression data independent of clinical features to predict the likelihood of HGPCa / GG2 on a subsequent needle biopsy.

## Background

Prostate cancer (PCa) is a leading cause of cancer death among men in the United States, with more than 3.6 million men living with prostate cancer. It is estimated that 174,650 newly diagnosed cases occurred in 2019 [1]. Prostate needle biopsies are typically recommended for men with elevated serum PSA levels and/or a suspicious digital rectal exam (DRE) with added considerations based on family history, age, and race. The anxiety, pain, and potential complications associated with prostate biopsy are well documented [2–6]. Furthermore, a large percentage of newly diagnosed prostate cancers are indolent, clinically insignificant, and with low metastatic potential. These cancers typically do not require definitive treatment and may be managed most effectively with Active Surveillance (AS). The low specificity of PSA which contributes to the high frequency of newly-diagnosed low-risk PCa suggests that 60–70% of men may be able to avoid biopsy [7–10]. Compounding the challenge, the majority of initial biopsy tests do not find cancer [11]. For each negative initial biopsy, it is unknown whether elevated PSA levels and/or a suspicious DRE facilitated the biopsy decision or if the biopsy simply missed a cancerous lesion due to sampling error, tumor heterogeneity and multifocality [12].

The European Randomized Study of Screening for Prostate Cancer (ERSPC) demonstrated that the overdiagnosis and overtreatment of PCa resulting from current practice requires more contemporary and improved methods to identify high-grade disease [13, 14]. Novel diagnostics such as the current test that provide additional clinical value to risk stratify high-grade PCa (Gleason Grade, or GG ≥2) have been developed with a primary objective to avoid biopsies for patients with an increased likelihood of having benign or non-aggressive (GG ≤1) disease [15–17].

The ExoDx Prostate assay relies on the isolation and analysis of urinary exosomes, which are small lipid bilayer membrane extra-cellular microvesicles (typically 30–200 nm in diameter) that are secreted from all living cells. Exosomes contain RNA, DNA, and protein, and have been identified in a variety of biofluids such as blood, cerebrospinal fluid, and urine. They are particularly promising for RNA expression profiling given their protected microanatomic environment [18–21]. The ExoDx Prostate (EPI) test has been extensively validated in two prospective multi-center US studies for biopsy naive patients and the signature is generated from quantitative analysis of PCA3 (prostate cancer antigen 3), ERG (V-ets erythroblastosis virus E26 oncogene homologs) and SPDEF RNA [16, 17].

The present analysis was designed to evaluate the performance of the ExoDx Prostate gene signature in a cohort of men with a history of prior negative biopsy and now presenting for a repeat biopsy.

## Methods

### Study population

First-catch, non-DRE urine samples were collected at 22 clinical sites (academic and community) in the USA from men with a prior negative prostate biopsy scheduled for a repeat biopsy between June 2014 and April 2015. The men were prostate cancer free, 50 years or older, and undergoing repeat biopsy for either a suspicious DRE and/or PSA level between 2 and 10 ng/mL. Men with a history of invasive treatment for benign prostatic hyperplasia within 6 months or taking medications that affect serum PSA levels within 3–6 months were excluded. The prostate biopsies were reported by the local hospital/practice pathologist who was blinded to ExoDx Prostate results. The study protocol was approved by the Western Institutional Review Board, Olympia, WA and individual academic institutional review boards (Johns Hopkins Hospital, University of Michigan); all study participants provided written informed consent and were not compensated for participating in the study.

### Sample collection and processing

First catch urine samples (25–50 mL) were stored at 4 °C for up to 14 days before shipment on ice to the central laboratory (Exosome Diagnostic Laboratory, Waltham, MA). At the Exosome Diagnostics CLIA Laboratory, samples were filtered (0.8um) and stored at -80 °C until further processing which included exosome isolation and concentration by ultrafiltration centrifugation.

For each sample, exosomal RNA was extracted, and the RNA copy numbers of ERG, PCA3, and SPDEF determined. The methodology for urinary exosome isolation, primer generation, RNA extraction and normalization as well as reverse transcriptase polymerase chain reaction have been previously reported in an original analytic and technical feasibility study by Donovan et al., 2015 [14] and in two subsequent independent validation studies [15, 16] of which the McKiernan et al., 2016 [15] provided the prior negative patients included in the current analysis. The test result is calculated based on the relative gene expression of these three genes, without inclusion of other clinical parameters, and provides a risk score (scale 0–100) that predicts the presence of HGPCa (≥GG2). Men with a score ≥ 15.6 (or 20) are at increased risk for having HGPCa on a subsequent biopsy.

### Statistical analysis

Evaluate ability of the EPI assay to predict GG 2 or higher prostate cancer (PCa) on repeat biopsy in men with a history of a prior negative biopsy and a PSA level 2–10 ng/mL. Area under the Receiver operating characteristics (AUC-ROC) curves for PSA vs. the EPI test are utilized as a measure of clinical performance. A well-established risk calculator (RC), The European Randomized Study of Screening for Prostate Cancer (ERSPC) RCs was utilized to further determine risk of GG2 or higher PCa using available clinical parameters [14, 22]. As prostate volume is not generally obtained, the ERSPC RC DRE is used as a surrogate for prostate volume [23]. Missing DRE results were imputed as nonsuspicious. The initial biopsy 15.6 cut point from two prior prospective studies [16, 17] was utilized in the repeat biopsy setting and compared to the EPI cut points of 20 (adjusted cutpoint from the first validation study [16] and 29.6 (adjusted cutpoint), using sensitivity, specificity, negative predictive value (NPV), and positive predictive value (PPV). EPI test was also assessed with a decision curve analysis to investigate the net health benefit for predicting GG2 or higher PCa [24]. The datasets analyzed during the current study may be available from the corresponding author on reasonable request.

## Results

### Study population and biopsy outcome

Between June 2014 and April 2015, non-DRE, first catch urine samples were collected from 1563 participants enrolled in a prospective validation study of the ExoDx Prostate test for men undergoing initial diagnostic prostate biopsy with a PSA between 2 and 10 ng/mL. The enrollment included patients with a prior negative biopsy however they were excluded in the validation analysis by design. Here we show the outcome in the population that had at least one prior negative biopsy. Urine samples from subjects who had incomplete data and/or > 49 ml were excluded of which all were initial biopsy patients. There were no patients excluded from the prior negative biopsy cohort. 229 patients met the criteria for the ‘Intended Use Population’: a prior negative biopsy and “gray zone” serum PSA levels (2–10 ng/mL). The median age was 65 years, and median pre-biopsy serum PSA was 6.1 ng/mL (Table 1). Most subjects had no family history of PCa (75.1%) and 66.4% had a non-suspicious DRE with 71.6% of subjects of Caucasian descent and 14.4% African American. Of the 229 patients, 90% underwent a 12-core, transrectal ultrasound-guided prostate needle biopsy using a standard template with diagnosis performed at site affiliated pathology practices without a central pathology review. The total positive biopsy rate was 31% (*n* = 72): 19% (*n* = 44) GG1 and 12% (*n* = 28) ≥ GG2 with 69% benign biopsies.

**Table 1:** Demographic and clinical characteristics of prior negative biopsy cohort

Total Cohort, N	229
Median Age (IQR)	65 (60–70)
Median PSA ng/mL (IQR)	6.1 (4.71–7.5)
Family History, N (%)	172 (75.1%)
Ethnicity, N (%)	
African Americans	33 (14.4%)
Asian/Pacific Islander	4 (1.7%)
Caucasian	164 (71.6%)
Hispanic	17 (7.4%)
Other	11 (4.8%)
DRE non-suspicious, N (%)	152 (66.4%)
Grade Groups, N (%)	
Benign	157 (68.6%)
GG1	44 (19.2%)
GG2	17 (7.4%)
GG3	6 (2.6%)
GG4	4 (1.4%)
GG5	1 (0.4%)

### EPI PERFORMANCE IN PREDICTING ≥GG2 PCa

In 229 men undergoing repeat biopsy for a prior negative biopsy, the ExoDx Prostate test with the prior validated cut point of 15.6 (for the initial biopsy) demonstrated good performance in discriminating HGPCa, GG2 or higher from low-grade prostate cancer (Gleason 6, GG1) and benign disease on biopsy. The assay performance in the prior negative patients showed an NPV and sensitivity of 92% (95%CI, 0.81–0.97) 82%, respectively. An EPI score less than or equal to 15.6 would have avoided 26% of all biopsies (i.e. 59 of 229) or 27% of unnecessary biopsies (i.e. true negative or specificity) and delayed detection of ≥ GG2 disease in 5 men (2.1%) (Table 2). Importantly, only 3 men with GG3 or higher (1%) would have delayed detection in the repeat biopsy setting. Using an EPI score of ≤20 or ≤ 29.6 would have avoided biopsies in 35% (i.e. 80 of 229) and 61% (i.e. 140 of 229) or 37 and 65%, respectively of unnecessary biopsies, thereby reducing anxiety and potential complications associated with an unnecessary biopsy. It is important to keep in mind that with the cut-point adjustments other measures of accuracy and performance will change, notably applying a cut-point of 29.6 will decrease the sensitivity to 68% while using either the 15.6 or 20 will yield comparable NPV and sensitivity while increasing the number of unnecessary biopsies from 26%^ to 37% with the 20 cut-point.

**Table 2:** Projected impact of the validated EPI 15.6 and adjusted cut-points 20 and 29.6 (with presumed 100% compliance) on expected biopsies performed and avoided

EPI cut point	Expected Biopsies Performed	Expected Biopsies Avoided	Delayed GG2	Delayed ≥GG3	NPV	Sensitivity	Specificity
15.6	170 (74%)	59 (26%)	2	3	91.5 (81.3–97.2)	82.1 (63.1–93.9)	26.9 (20.9–33.6)
20	149 (65%)	80 (35%)	2	3	93.8 (86.0–97.9)	82.1 (63.1–93.9)	37.3 (30.6–44.4)
29.6	89 (39%)	140 (61%)	2	6	93.6 (88.1–97.0)	67.9 (47.6–84.1)	65.2 (58.2–71.7)

**Fig. 1:**
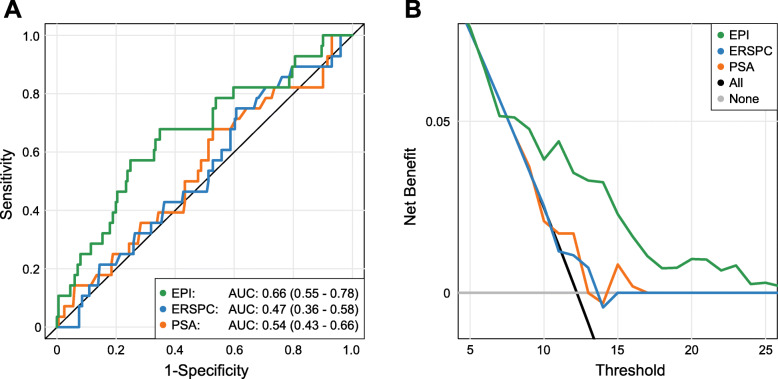
Area under receiver operating characteristic (AUC) curves are shown to compare performances of EPI in (**a**) the current cohort (*n* = 229) with the ERSPC, and PSA alone. The corresponding net benefit analysis for this cohort is shown in **b**. EPI = ExoDx Prostate (IntelliScore); ERSPC = European Randomized Study of Screening for prostate cancer (risk calculator); PSA = prostate-specific antigen

On comparing the performance of the EPI test (AUC 0.66, 95%CI: 0.55–0.78) with alternative models, the EPI test was superior to PSA only (AUC 0.54, 95%CI: 43–66), and the ERSPC-RC (AUC 0.47, 95%CI: 0.36–0.58), for predicting ≥GG2 PCa, (Fig. 1a).

We also investigated the clinical value of the EPI test relative to alternative models using a decision curve analysis (Fig. 1b). EPI’s performance was superior (highest net benefit) to all models tested, including PSA and ERSPC-RC.

## Discussion

Over 1 million transrectal ultrasonography guided (TRUS) prostate biopsies are performed annually in the US and Europe [6]. Although clinical information such as age, race, family history, or a suspicious DRE are triggers, most TRUS biopsies are driven by PSA screening. Biopsies are often associated with pain, bleeding, sepsis and, of significant concern, an increasing rate of antibiotic resistant infection [6, 25]. A significant number (approximately 70% of men) are not found to have prostate cancer on initial biopsy, and this leads to patient anxiety because of the false negative rate as a result of prostatectomy under-sampling and tumor heterogeneity/multifocality. These concerns drive many men to undergo repeat biopsy. In fact, the Surveillance, Epidemiology, and End Results (SEER) data indicates that ~ 12% of men with a prior negative biopsy have a repeat biopsy within 1 year and 44% of men younger than 70 years old have a repeat biopsy [26].

The ExoDx (EPI) Prostate test is a urine-based genomic assay which uses the expression levels of three genes (ERG, PCA3 and SPDEF) involved in PCa initiation and progression, to predict risk of HGPCa, independent of clinical features or standard of care (SOC) [27–33].. The test algorithm was developed and validated on the intended use cohort, i.e. men presenting for their initial biopsy with a PSA 2–10 ng/mL where it achieved an NPV of 91%, a sensitivity of 91 and 34% specificity [16]. A recent blinded control arm utility study demonstrated that the EPI test reduced the number of biopsies when the test was negative, and helped to identify 30% more high-grade prostate cancers compared to the control arm without the EPI test result [34]. It is well established in the literature that prevalence of prostate cancer is significantly lower in a prior negative biopsy population and that this clinical feature will favorably impact test performance when included in risk assessment models (and some commercial assays) [35, 36]. To address this issue, we evaluated the accuracy of EPI in the prior negative biopsy population using the AUC and compared results with PSA alone and a well-established risk calculator, the European Randomized Study of Screening for Prostate Cancer (ERSPC) [13, 14]. EPI had a superior AUC of 0.66 vs PSA AUC 0.54, and ERSPC AUC 0.47. As EPI is independent of all clinical features it represents an independent biological assessment of prostate cancer risk.

In addition to the AUC we also employed a decision curve analysis which considers overall net health benefit of various biomarkers and risk assessment tools. When EPI was compared with PSA and clinical-only models such as the ERSPC, a higher net benefit was observed at a biopsy threshold probability of 10%, and maintained up to a maximum biopsy rate of 30%. In contemporary practice the decision to proceed with a biopsy is a delicate balance between the risk associated with the biopsy procedure vs. the risk of missing a potentially clinically significant cancer. The benefit for a test such as EPI is that the risk score is a biological construct independent of all clinical factors thereby allowing for combination with other variables such as race, family history and an underlying disease.

In the current prior negative biopsy population, an EPI risk score less than 15.6 would have potentially avoided biopsy in 26% of men based on total number of biopsies in the study (i.e. 229) and a score of less than 20 would have avoided a biopsy in 35% of men without missing additional GG2 or higher PCa. Furthermore, if we equate benign and Grade Group 6 biopsy outcomes as unnecessary biopsies, the < 15.6 and < 20 cut-points would represent 27 and 37% unnecessary biopsies, respectively. Furthermore, as per the 2020 NCCN Early Detection of Prostate Cancer Guidelines [37], it is clinically important to differentiate GG2 vs GG3 PCa which is relevant for the performance of EPI as only 3 patients with GG3 or higher disease were missed at either the 15.6 or the 20 cut-point vs. 6 individuals (3%) with the 29.6 cut-point. The EPI assay also generated a comparable NPV of 92 and 94% in the prior negative biopsy population (cut-points 15.6 and 20, respectively) vs. 91 and 89% in the two prior initial biopsy validation studies [16, 17].

Study limitations include the sample size and lack of a central pathology review, which may have introduced some variability, specifically when reporting small volume cancers and the fact that there were no men in the study who underwent multi-parametric MRI imaging pre-biopsy. During the study period (2014 and 2015), MRI imaging was not standard of care in the USA. Nevertheless, all the study participating centers represent large urology group practices and academic centers with highly experienced uropathologists. A second prospective study in prior negative biopsy men is underway in the US to address the increasing use of mpMRI imaging and fusion biopsy in this population.

## Conclusion

The ExoDx Prostate *(IntelliScore)* EPI test is a validated, non-invasive urine exosome gene expression assay that performs equally well for men at initial biopsy or with a history of at least one prior negative biopsy. The gene expression assay is more accurate than existing risk assessment methods, is not dependent on clinical features, and informs decision-making at both initial and or repeat biopsy time-points.

## Data Availability

The datasets during and/or analyzed during the current study may be available from the corresponding author on reasonable request.

## Abbreviations

PCA3: Prostate cancer antigen 3
ERG: ETS (erythroblast transformation-specific) -related gene
SPDEF: SAM pointed domain-containing ETS transcription factor
